# Supplementary material for: An increase of NPY1 expression leads to inhibitory phosphorylation of PIN-FORMED (PIN) proteins and suppression of pinoid (pid) null mutants
Source: eLife. 2025 Dec 17;14:RP108207. doi: 10.7554/eLife.108207 (PMC12711196; doi:10.7554/eLife.108207)
Supplement: Supplementary file 1. — NPY1 overexpression T1 plants with pid-c1+/- genotype was self-pollinated. At the T2 stage, three genotypes were selected: WT without transgenes and without pid-c1 mutation (this is called WT-xx, xx refers to the line number); NPY1 OE without pid-c1 mutation (called NPY1 in WT xx); NPY1 OE in pid c1 (called NPY1 in pid xx). Inflorescence heads with flower buds of the lines were used for proteomic analysis. All of the detected NPY1 peptides were more abundant in the overexpression lines than in WT. The two most abundant peptides in NPY1 OE lines were highlighted in yellow. PID did not affect NPY1 phosphorylation, as all of the NPY1 phospho-peptides were detected in pid-c1 background. [file elife-108207-supp1.docx]

| **NPY1 Peptides** | **NPY1 OE in**  **WT 68/WT-68** | **NPY1 OE in WT 83//WT-83** | **Average** | **p-value** | **NPY1 OE in *pid* 68/WT-68** | **NPY1 OE in *pid* 83/WT-83** | **Average** | **p value** |
| --- | --- | --- | --- | --- | --- | --- | --- | --- |
| ILVNPETITWSYTFNR | 2.35559248 | 2.4135503 | 2.38457139 | 1.98E-05 | 2.21619975 | 2.35693343 | 2.28656659 | 5.56E-05 |
| ANHSPVASVAASSHSPVEK | 15.7900748 | 15.7282527 | 15.7591638 | 3.47E-05 | 9.64734912 | 14.5077545 | 12.0775518 | 0.00028 |
| ANHSPVASVAASSHSPVEK | 2.67605261 | 2.61597069 | 2.64601165 | 4.33E-05 | 2.08450455 | 2.19393103 | 2.13921779 | 0.00013303 |
| SGGGAQLMPSR | 5.86865743 | 5.28826611 | 5.57846177 | 6.27E-05 | 3.94201085 | 4.5143918 | 4.22820132 | 0.00032349 |
| ANHSPVASVAASSHSPVEK | 3.25636105 | 3.69909646 | 3.47772875 | 9.22E-05 | 2.79779869 | 3.20416118 | 3.00097994 | 0.00010021 |
| SSEVSSGSSQSPPAK | 1.90655777 | 2.09629989 | 2.00142883 | 0.00015918 | 1.75657155 | 1.95325049 | 1.85491102 | 0.00033173 |
| ANHSPVASVAASSHSPVEK | 7.52570342 | 8.72233498 | 8.1240192 | 0.00078166 | 7.33410315 | 5.57817461 | 6.45613888 | 0.00056868 |
| ANHSPVASVAASSHSPVEK | 4.02351215 | 4.49813655 | 4.26082435 | 0.00139384 | 4.23358793 | 3.30034108 | 3.76696451 | 0.00073176 |
| ANHSPVASVAASSHSPVEK | 3.76376127 | 3.9373259 | 3.85054358 | 0.00152686 | 3.94591283 | 2.88089402 | 3.41340343 | 0.00133111 |
| ANHSPVASVAASSHSPVEK | 22.737353 | 21.7293264 | 22.2333397 | 0.00164477 | 22.9081916 | 15.4378037 | 19.1729976 | 0.00031642 |
| ANHSPVASVAASSHSPVEK | 1.86599246 | 2.0258777 | 1.94593508 | 0.00267761 | 1.58544666 | 1.96489964 | 1.77517315 | 0.00531702 |
| ANHSPVASVAASSHSPVEK | 3.88225666 | 3.52063452 | 3.70144559 | 0.00277971 | 4.08073192 | 2.65260553 | 3.36666872 | 0.00306056 |
| ANHSPVASVAAssHSPVEK | 1.75264424 | 1.91621441 | 1.83442933 | 0.00348417 | 1.42846438 | 1.88351342 | 1.6559889 | 0.00868568 |
| ANHSPVASVAASSHSPVEK | 2.41617929 | 2.98021238 | 2.69819583 | 0.00518181 | 2.66996976 | 2.15429695 | 2.41213335 | 0.00151103 |
| LHEASVK | 2.06468683 | 1.73796425 | 1.90132554 | 0.01196239 | 2.02413175 | 1.8241927 | 1.92416223 | 0.0009437 |

**Supplementary file 1. Phospho-peptides from NPY1 protein in both WT and *pid-c1* backgrounds**. *NPY1* overexpression T1 plants with *pid-c1*+/- genotype was self-pollinated. At T2 stage, three genotypes were selected: WT without transgenes and without *pid-c1* mutation (this is called WT-xx, xx refers to the line number); NPY1 OE without *pid-c1* mutation (Called NPY1 in WT xx); NPY1 OE in *pid c1* (called NPY1 in *pid* xx). Inflorescence heads with flower buds of the lines were used for proteomic analysis. All of the detected NPY1peptides were more abundant in the overexpression lines than in WT. Two most abundant peptides in NPY1 OE lines were highlighted yellow. PID did not affect NPY1 phosphorylation, as all of the NPY1 phospho-peptides were detected in *pid-c1* background.
