# Supplementary material for: An increase of NPY1 expression leads to inhibitory phosphorylation of PIN-FORMED (PIN) proteins and suppression of pinoid (pid) null mutants
Source: eLife. 2025 Dec 17;14:RP108207. doi: 10.7554/eLife.108207 (PMC12711196; doi:10.7554/eLife.108207)
Supplement: Supplementary file 2. — L4 and L35 refer to NPY1∆C OE lines #4 and #35, respectively. WT: wild type. Lower case s or t in the peptides refers to the phosphorylated residues. The PIN2 phospho-peptides are highlighted in yellow, which were enriched in the NPY1∆C OE lines. [file elife-108207-supp2.docx]

| **Peptide** | **Protein** | **L4/WT** | **L4/WT p-value** | **L35/WT** | **L35/WT p-value** |
| --- | --- | --- | --- | --- | --- |
| AGLQVDNGANEQVGKsDQGGAK | PIN7 | 6.2 | 1.2609E-06 | 3.8 | 4.860007E-06 |
| AGLNVFGGAPDNDQGGRsDQGAK | PIN3 | 7.7 | 1.0026E-07 | 5.2 | 5.964452E-07 |
| MLVPDQsHNGETK | PIN3 | 1.8 | 3.1218E-04 | 2.7 | 5.402182E-05 |
| AVAHPAS GDFGGEQQFSFAGK | PIN3 | 2.4 | 3.2374E-06 | 2.3 | 1.016313E-05 |
| TGLGGAEASQRK | PIN3 | 2.3 | 3.8482E-05 | 2.1 | 3.783133E-05 |
| LAPNSTAALQSK | PIN3 | 1.4 | 6.6915E-03 | 1.9 | 4.228866E-04 |
| AVAHPAS GDFGGEQQFSFAGK | PIN3 | 2.2 | 8.9864E-07 | 1.8 | 1.769637E-06 |
| AVAHPASGDFGGEQQFSFAGK | PIN3 | 1.4 | 9.9089E-05 | 1.3 | 3.237153E-05 |
| VLATDGGNNISNKtTQAK | PIN1 | 2.4 | 1.6854E-05 | 3.0 | 6.027058E-05 |
| NsNFGPGEAVFGSK | PIN1 | 1.2 | 1.9247E-04 | 1.5 | 1.909081E-05 |
| ISVPQGNSNDNQYVER | PIN1 | 1.8 | 3.1823E-05 | 1.4 | 5.275605E-04 |
| AAPQATAAGGGASMEEGAAGK | PIN6 | 1.5 | 1.9891E-03 | 1.8 | 2.622491E-04 |
| KsGGDDIGGLDSGEGER | PIN4 | 3.4 | 5.4516E-06 | 2.8 | 1.602179E-05 |
| SGGDDIGGLDSGEGEREIEK | PIN4 | 0.7 | 5.8252E-03 | 1.2 | 4.216364E-03 |
| GGGDDIGGLDSGEGEREIEK | PIN4 | 4.6 | 2.4332E-06 | 8.0 | 1.574718E-07 |
| ANHSPVASVAAS SHSPVEK | NPY1 | 8.4 | 9.6544E-06 | 18.7 | 6.380869E-08 |
| ANHSPVAS VAASSHSPVEK | NPY1 | 5.8 | 2.1392E-06 | 12.9 | 7.023571E-08 |
| SGGGAQLMPSRSR | NPY1 | 3.8 | 6.8117E-06 | 12.7 | 4.261671E-07 |
| ANHSPVASVAASSHSPVEK | NPY1 | 4.3 | 1.3515E-03 | 10.2 | 1.110878E-04 |
| ANHSPVASVAAS SHSPVEK | NPY1 | 4.4 | 2.7638E-05 | 8.6 | 5.221852E-07 |
| SGGGAQLMPSR | NPY1 | 4.0 | 1.9647E-05 | 7.0 | 4.966188E-06 |
| ANHSPVAS VAAS SHSPVEK | NPY1 | 3.4 | 2.3040E-06 | 6.8 | 2.077026E-08 |
| ANHSPVASVAASsHSPVEK | NPY1 | 3.3 | 2.3647E-06 | 6.4 | 4.733227E-09 |
| ANHSPVASVAAS SHSPVEK | NPY1 | 3.1 | 2.1728E-04 | 6.0 | 7.799539E-07 |
| ANHSPVASVAASSHSPVEK | NPY1 | 2.3 | 1.7957E-05 | 3.3 | 7.890671E-07 |
| ANHSPVASVAASSHSPVEK | NPY1 | 1.9 | 3.3120E-05 | 3.2 | 8.332437E-08 |
| ANHSPVASVAASSHSPVEK | NPY1 | 1.9 | 6.5198E-04 | 3.0 | 3.798516E-05 |
| ANHSPVAS VAASSHSPVEK | NPY1 | 2.0 | 6.7311E-05 | 2.8 | 3.136031E-05 |
| ANHSPVAS VAASSHSPVEK | NPY1 | 1.8 | 7.4950E-04 | 2.7 | 1.700945E-04 |
| ANHSPVAS VAAS SHSPVEK | NPY1 | 1.7 | 1.3890E-03 | 2.6 | 8.815327E-05 |
| ANHSPVASVAAss HSPVEK | NPY1 | 1.5 | 1.9497E-03 | 2.3 | 1.001435E-05 |
| ANHSPVAS VAASS HSPVEK | NPY1 | 1.6 | 4.3496E-05 | 2.2 | 4.463782E-06 |
| ANHSPVASVAASSHSPVEK | NPY1 | 1.4 | 1.5319E-02 | 1.6 | 2.684608E-03 |
| ANNNGSSSTGNSTPEVIPASR | NPY5 | 1.3 | 1.4448E-02 | 1.2 | 1.918117E-02 |
| ESGGGGGGGVGVGGQNK | PIN2 | 4.2 | 3.1309E-04 | 5.6 | 1.631471E-04 |
| KGSDVEDGGPGPR | PIN2 | 3.5 | 6.0552E-06 | 3.7 | 3.433063E-06 |
| HGYTNSYGGAGAGPGGDVYSLQSSK | PIN2 | 2.2 | 1.4621E-03 | 2.2 | 1.781404E-04 |

**Supplementary file 2. Phosphorylation of NPY1 and PIN proteins caused by *NPY1∆C* overexpression**. L4 and L35 refer to *NPY1∆C OE* line #4 and #35, respectively. WT: wild type. Lower case s or t in the peptides refers to the phosphorylated residues. The PIN2 phospho-peptides are highlighted in yellow, which were enriched in the *NPY1∆C OE* lines.
