# Supplementary material for: An increase of NPY1 expression leads to inhibitory phosphorylation of PIN-FORMED (PIN) proteins and suppression of pinoid (pid) null mutants
Source: eLife. 2025 Dec 17;14:RP108207. doi: 10.7554/eLife.108207 (PMC12711196; doi:10.7554/eLife.108207)
Supplement: Supplementary file 3. [file elife-108207-supp3.docx]

**Supplementary File 3. Primers used in this study**

| Primer Name | Primer Sequence | Description |
| --- | --- | --- |
| UBQ-NPY1-F | CTGAGTTTTTCTGATTAACAGCTCATGAAGTTCATGAAGCTAGGGTCTAAG | Primers for amplifying the NPY1 to clone into UBQ-NPY1 |
| UBQ-NPY1-R | CTGGGAGGCCTGGATCGATATCAATTTCACGATATCGAATGTCTGCGG |  |
| UBQ-NPY1-C-R | CTGGGAGGCCTGGATCGATATCAATTTCAGTTGCTAATCTCTCCTTTCCCAG | Alternate reverse primer to omit the C-terminus of NPY1 |
| UBQ-MfeI-5P | GCTGCAGGTCGACGCGTCAATTCGACGAGTCAGTAATAAACG | Sequencing primer |
| UBQ-MfeI-3P-RC | CTGGGAGGCCTGGATCGATATCAATTGAGCTGTTAATCAGAAAAACTCAG | Primers for cloning the AtUBQ10 promoter into the MfeI site of pHDE |
| PIN1-SGT1 | TCTTTGAGTACCGTGGAGCTAAGC | Genotyping pin1 mutants |
| PIN1-SGT2 | GCCACCACTTCCTCCAGATTGATA | Genotyping pin1 mutants |
| PIN1-S-GT3 | CAGGTGATGCCGAATAAACTGGA | Genotyping pin1 mutants |
| PIN1-SGT4 | ATCTTCACACCAGACCAATGCTCC | Genotyping pin1 mutants |
| PIN1-sGT5 | TCCACCGCTACGAACGATCATCA | Genotyping pin1 mutants |
| PIN1-GFP-out1 | GTCGGAACTCTAACTTTGGTCCT | PIN1-GFP-out1 + PIN1-GFP-out2 for determining zygosity of PIN1-GFP HDR |
| PIN1-GFP-out2 | ATAAGCAGAGAACTGTGGAGCAT | PIN1-GFP-out1 + PIN1-GFP-out2 for determining zygosity of PIN1-GFP HDR |
| PIN1-GFP-IN-1 | GACGGTGGGAACAACATAAGCA | With PIN1-GFP-out2, determines the presence of GFP insertion |
| PIN1-GFP-IN-2 | GAGTCTTGTCATCACACTTGTTGG | with PIN1-GFP-out1, determines the presence of GFP insertion |
| PID-KO-RP2 | cgaacgccgctggtttg | Genotyping pid-TD mutants |
| PID-KO-LP2 | gatgttacgagaatcagacg | Genotyping pid-TD mutants |
| JMLB1 | GGCAATCAGCTGTTGCCCGTCTCACTGGTG | Genotyping pid-TD mutants |
| PID-NGT1 | Gaagagagatcatgcaactggtcgg | Genotyping pid-c mutant |
