# Supplementary material for: An increase of NPY1 expression leads to inhibitory phosphorylation of PIN-FORMED (PIN) proteins and suppression of pinoid (pid) null mutants
Source: eLife. 2025 Dec 17;14:RP108207. doi: 10.7554/eLife.108207 (PMC12711196; doi:10.7554/eLife.108207)
Supplement: Supplementary file 4. [file elife-108207-supp4.docx]

Supplementary file 4. TMT labelling schemes

| TMTpro | 126 | 127N | 127C | 128N | 128C | 129N | 129C | 130N | 130C | 131N | 131C | 132N |
| --- | --- | --- | --- | --- | --- | --- | --- | --- | --- | --- | --- | --- |
| Samples | WT  68-1 | WT  68-2 | *NPY1*  *OE* in  WT  68-1 | *NPY1*  *OE* in  WT-  68- 2 | *NPY1*  *OE* in *pid-c1*  68-1 | *NPY1*  *OE* in *pid-c1*  68-2 | WT  83-1 | WT  83-2 | *NPY1*  *OE* in  WT  83-1 | *NPY1*  *OE* in  WT  83-2 | *NPY1*  *OE* in *pid-c1*  83-1 | *NPY1*  *OE* in *pid-c1*  83-2 |

| TMT | 126 | 127N | 127C | 128N | 128C | 129N | 129C | 130N | 130C |
| --- | --- | --- | --- | --- | --- | --- | --- | --- | --- |
| NPY1*∆C* Samples | WT-1 | WT-2 | WT-3 | L4-1 | L4-2 | L4-3 | L35-1 | L35-2 | L35-3 |
