# Supplementary material for: An increase of NPY1 expression leads to inhibitory phosphorylation of PIN-FORMED (PIN) proteins and suppression of pinoid (pid) null mutants
Source: eLife. 2025 Dec 17;14:RP108207. doi: 10.7554/eLife.108207 (PMC12711196; doi:10.7554/eLife.108207)
Supplement: Peer review history

# Editor report: eLife Assessment

**DOI:** 10.7554/eLife.108207.2.sa0

**Authors:** Detlef Weigel

**Affiliations:** https://ror.org/0243gzr89 Max Planck Institute for Biology Tübingen Germany

**Keywords:** Solid, Valuable

This **valuable** study concerns a highly interesting and biologically relevant topic, the regulation of the PIN auxin transporter, which is of broad interest to the plant biology community. The authors propose NPY1 to act downstream of PID in auxin-mediated development by modulating PIN phosphorylation, which, if experimentally solidified, would expand our understanding of PIN regulation. While the genetic evidence is **solid**, the mechanistic role of NPY1 and the functional relevance of phosphorylated PIN residues are still uncertain. There are also concerns regarding experimental rigor and methodological transparency.

# Referee report: Reviewer #1 (Public review)

**DOI:** 10.7554/eLife.108207.2.sa1

Summary:

The authors of this study propose a model in which NPY family regulators antagonize the activity of the pid mutation in the context of floral development and other auxin-related phenotypes. This is hypothesized to occur through regulation of or by PID and its action on the PIN1 auxin transporter.

Strengths:

The findings are intriguing.

Weaknesses and Major Comments:

(1) While the findings are indeed intriguing, the mechanism of action and interaction among these components remains poorly understood. The study would benefit from significantly more thorough and focused experimental analyses to truly advance our understanding of pid phenotypes and the interplay among PID, NPYs, and PIN1.

(2) The manuscript appears hastily assembled, with key methodological and conceptual details either missing or inconsistent. Although issues with figure formatting and clarity (e.g., lack of scale bars and inconsistent panel layout) may alone warrant revision, the content remains the central concern and must take precedence over presentation.

(3) Given that fertile progeny are obtained from pid-TD pin1/PIN1 and pid NPY OE lines, it would be important to analyze whether mutations and associated phenotypes are heritable. This is especially relevant since CRISPR lines can be mosaic. Comprehensive genotyping and inheritance studies are required.

(4) The Materials and Methods section lacks essential information on how the lines were generated, genotyped, propagated, and scored. There is also generally no mention of how reproducible the observations were. These genetic experiments need to be described in detail, including the number of lines analyzed and consistency across replicates.

(5) The nature of the pid alleles used in the study is not described. This is essential for interpretation.

(6) The authors measure PIN1 phosphorylation in response to NPY overexpression and conclude that the newly identified phosphorylation sites are inhibitory because they do not overlap with known activating sites. This conclusion is speculative without functional validation. Functional assays are available and must be included to substantiate this claim.

(7) Figure 5 implies that NPY1 acts downstream of PID, but there is no biochemical evidence supporting this hierarchy. Additional experiments are needed to demonstrate the epistatic or regulatory relationship.

(8) The authors should align their genetic observations with cell biological data on PIN1, PIN2, and PID localization and distribution.

# Referee report: Reviewer #2 (Public review)

**DOI:** 10.7554/eLife.108207.2.sa2

Summary:

The study is well-conducted, revealing that NPY1, with previously less-characterized molecular functions, can suppress pid mutant phenotypes with a phosphorylation-based mechanism. Overexpression of NPY1 (NPY1-OE) results in PIN phosphorylation at unique sites and bypasses the requirement for PID for this event. Conversely, a C-terminal deleted form of NPY1 (NPY1-dC) fails to rescue pid despite promoting a certain phospho-profile in PIN proteins.

Strengths:

(1) The careful genetic analyses of pid suppression by NPY1-OE and the inability of NPY1dC to do the same.

(2) Phospho-proteomics approaches reveal that NPY1-OE induces phosphorylation of PINs at non-canonical sites, independent of PID.

Weaknesses:

(1) The native role of NPY1 is not tested by phospho-proteomics in loss-of-function npy1 mutants. Such analysis would be crucial to demonstrate that NPY1 is required for the observed phosphorylation events.

(2) The functional consequences of the newly identified phosphorylation sites in PINs remain speculative. Site-directed mutagenesis (phospho-defective and phospho-mimetic) would help clarify their physiological roles.

(3) The kinase responsible for NPY1-mediated phosphorylation remains unidentified. Since NPY1 is a non-kinase protein, a model involving recruitment of partner kinases (e.g., PIN-phosphorylating kinases other than PID) should be considered or discussed.

# Referee report: Reviewer #3 (Public review)

**DOI:** 10.7554/eLife.108207.2.sa3

Summary:

This manuscript from Mudgett et al. explores the relative roles of PID and NPY1 in auxin-dependent floral initiation in Arabidopsis. Micro vectorial auxin flows directed by PIN1 are essential to flower initiation, and loss of PIN1 or two of its regulators, PID and NPY1 (in a yucca-deficient background) phenocopies the pinformed phenotype. This group has previously shown that PID-PIN1 interactions and function are dosage-dependent. The authors pick up this thread by demonstrating that a heterozygote containing a CRISPR deletion of one copy of PIN1 can restore quasi-wild type floral initiation to pid.

The authors then show that overexpression of NPY1 is sufficient to more or less restore wild-type floral initiation to the pid mutant. The authors claim that this result demonstrates that NPY1 functions downstream of PID, as this ectopic abundance of NPY1 resulted in phosphorylation of PIN1 at sites that differ from sites of action of PID. The authors pursue evidence that PID action via NPY1 is analogous to the mode of action by which phot1/2 act on NPH3 in seedling phototropism. Such a model is supported by the evidence presented herein that the C terminus of NPY1, which has abundant Ser/Thr content, is phosphorylated, and that the deletion of this domain prevents overexpression compensation of the pinformed phenotype.

While the results presented support evidence in the literature that PID acts on NPY1 to regulate PIN1 function, it is also possible that NPY1 overexpression results in limited expansion of phosphorylation targets observed with other AGC kinases. And if the phot model is any indication, there may be other PID targets that modulate PIN1-dependent floral initiation.

However, overexpression of the NPY1 C-terminal deletion construct resulted in phosphorylation of both PIN1 and PIN2 and agravitropic root growth similar to what is observed in pin2 mutants. This suggests that direct PID phosphorylation of PINs and action via NPY1 can be distinguished by phosphorylation sites and by growth phenotypes.

Strengths:

A very important effort that places NPY1 downstream of PID in floral initiation.

Weaknesses:

As PID has been shown to act on sites that regulate PIN protein polarity as well as PIN protein function, it would be useful if the authors consider how their results would fit/not fit with a model where combinatorial function of NPY1 and PID regulate PIN1 in a manner similar to the way that PID appears to function combinatorially with D6PK on PIN3.

# Author comment: Author response

**DOI:** 10.7554/eLife.108207.2.sa4

**Authors:** Michael Mudgett, Zhouxin Shen, Ruofan Kang, Xinhua Dai, Steven P Briggs, Yunde Zhao

**Affiliations:** University of California, San Diego La Jolla United States; University of California, San Diego La Jolla United States; University of California, San Diego La Jolla United States; University of California, San Diego La Jolla United States; University of California San Diego La Jolla United States; University of California, San Diego La Jolla United States

**Reviewer #1 (Public review):**

Summary:

The authors of this study propose a model in which NPY family regulators antagonize the activity of the pid mutation in the context of floral development and other auxin-related phenotypes. This is hypothesized to occur through regulation of or by PID and its action on the PIN1 auxin transporter.

Strengths:

The findings are intriguing.

We are pleased that the reviewer found the work interesting!

Weaknesses and Major Comments:

(1) While the findings are indeed intriguing, the mechanism of action and interaction among these components remains poorly understood. The study would benefit from significantly more thorough and focused experimental analyses to truly advance our understanding of pid phenotypes and the interplay among PID, NPYs, and PIN1.

Elucidating the mechanism of action and interaction among these components will require years of additional research. As key steps toward these goals, our work clearly established that (1) NPY1 functions downstream of PID, as overexpression of NPY1 completely suppressed *pid* phenotypes. This is surprising because the predominant model is that PID functions by directly phosphorylating and activating PINs without the need of NPY1 involvement. (2) In the absence of PID, NPY1 protein accumulated less in the NPY1 OE lines, suggesting that PID plays a role in affecting NPY1 stability/degradation/accumulation. We are not sure what are the exact experiments this reviewer is proposing.

Regarding *pid* phenotypes, *pid* is completely sterile in our conditions, while the suppression by NPY1 OE is very clear and the lines are fertile.

(2) The manuscript appears hastily assembled, with key methodological and conceptual details either missing or inconsistent. Although issues with figure formatting and clarity (e.g., lack of scale bars and inconsistent panel layout) may alone warrant revision, the content remains the central concern and must take precedence over presentation.

We did not include scale bars in our figures because the phenotype of interest is presence/absence of flowers. Readers should compare the mutants with the rescued plants and the WT plants.

(3) Given that fertile progeny are obtained from pid-TD pin1/PIN1 and pid NPY OE lines, it would be important to analyze whether mutations and associated phenotypes are heritable. This is especially relevant since CRISPR lines can be mosaic. Comprehensive genotyping and inheritance studies are required.

We only use stable, heritable, Cas9-free mutants in our studies. We genotype our mutants in every generation. More details have been added to the Materials and Methods section. We provide the genetic materials we use to the scientific community when requested to enable verification and extension of our results.

(4) The Materials and Methods section lacks essential information on how the lines were generated, genotyped, propagated, and scored. There is also generally no mention of how reproducible the observations were. These genetic experiments need to be described in detail, including the number of lines analyzed and consistency across replicates.

More details have been added to the Materials and Methods section

The criticism is not fully accurate. For example, we stated in the main text: “We genotyped T2 progenies from two *pid-c1* heterozygous T1 plants (#68 and # 83) for the presence of *pid*-*c1* and for *pid-c1* zygosity. We used mCherry signal, which was included in the *NPY1 OE* construct, as a proxy to determine the presence and absence of the *NPY1* transgene. For each line, we identified T2 plants without the *NPY1* transgene and without the *pid-c1* mutation (called WT-68 and WT-83, respectively). We also isolated T2 plants that contained the *NPY1* overexpression construct, but did not have the *pid*-c1 mutation (called *NPY1 OE* #68 in WT, and *NPY1 OE* #83 in WT). Finally, we identified T2 plants that were *pid-c1* homozygous and that had the *NPY1* transgene (called NPY1 OE #68 in *pid-c1* and NPY1 OE #83 in *pid-c1*). These genetic materials enabled us to compare the same *NPY1 OE* transgenic event in different genetic backgrounds.”

The genetic materials used are freely available to the scientific community. We would like to point out that we used several *pin1* and *pid* alleles to make sure that the phenotypes are caused by the genes of interest.

(5) The nature of the pid alleles used in the study is not described. This is essential for interpretation.

The mutants were described in a previous paper M. Mudgett, Z. Shen, X. Dai, S.P. Briggs, & Y. Zhao, Suppression of pinoid mutant phenotypes by mutations in PIN-FORMED 1 and PIN1-GFP fusion, Proc. Natl. Acad. Sci. U.S.A. 120 (48) e2312918120, https://doi.org/10.1073/pnas.2312918120 (2023). We have added the relevant information to Materials and Methods.

(6) The authors measure PIN1 phosphorylation in response to NPY overexpression and conclude that the newly identified phosphorylation sites are inhibitory because they do not overlap with known activating sites. This conclusion is speculative without functional validation. Functional assays are available and must be included to substantiate this claim.

We concluded that the phosphorylation of PINs in NPY1 OE is inhibitory on the basis of the following: (1) *pid* is suppressed in *pin*1 heterozygous backgrounds and by PIN1-GFP_HDR,_ demonstrating that partial loss of function of PIN1 or a decrease in *PIN1* gene dosage, which decreases PIN1 protein expression, caused the suppression of *pid*. (2) *pid* is completely suppressed by NPY1 OE, which caused an increase of PIN phosphorylation, suggesting that phosphorylation of PINs in NPY1 OE lines is inhibitory. It is true that we do not have biochemical data to support the conclusion. We would like to point out that the phosphorylation sites in PINs identified in this work do overlap with previously identified sites.

PIN activity assays are conducted in heterologous systems that do not include NPY proteins. Since NPY is important for PIN activities, we believe that these assays may provide misleading results. Moreover, PIN1 is likely part of a large protein complex. Without knowing the composition of the complex, functional assays in heterologous systems will not be interpretable.

(7) Figure 5 implies that NPY1 acts downstream of PID, but there is no biochemical evidence supporting this hierarchy. Additional experiments are needed to demonstrate the epistatic or regulatory relationship.

We show that overexpression of NPY1 completely suppressed the *pid* phenotype, and this epistatic relationship indicates that NPY1 functions downstream of PID. Moreover, we report that PID is required for NPY1 accumulation, indicating that PID is upstream of NPY1.

(8) The authors should align their genetic observations with cell biological data on PIN1, PIN2, and PID localization and distribution.

We are hesitating in using traditional PIN1-GFP, PIN2-GFP lines, as they are not stable in our hands. Localization of PID is still not clear. We have generated PID-GFP_HDR_ lines, but we could not detect any fluorescent signals (unpublished results). In addition, maize PINOID (BIF2) localizes to the nucleus, cytoplasm and cell periphery (Skirpan, A., Wu, X. and McSteen, P. (2008), Genetic and physical interaction suggest that BARREN STALK1 is a target of BARREN INFLORESCENCE2 in maize inflorescence development. The Plant Journal, 55: 787-797. https://doi.org/10.1111/j.1365-313X.2008.03546.x)

We would rather wait for the proper genetic materials before devoting our effort to this.

**Reviewer #2 (Public review):**

Summary:

The study is well-conducted, revealing that NPY1, with previously less-characterized molecular functions, can suppress pid mutant phenotypes with a phosphorylation-based mechanism. Overexpression of NPY1 (NPY1-OE) results in PIN phosphorylation at unique sites and bypasses the requirement for PID for this event. Conversely, a C-terminal deleted form of NPY1 (NPY1-dC) fails to rescue pid despite promoting a certain phospho-profile in PIN proteins.

Strengths:

(1) The careful genetic analyses of pid suppression by NPY1-OE and the inability of NPY1dC to do the same.

(2) Phospho-proteomics approaches reveal that NPY1-OE induces phosphorylation of PINs at non-canonical sites, independent of PID.

Thank you for having accurately summarized the main findings

Weaknesses:

(1) The native role of NPY1 is not tested by phospho-proteomics in loss-of-function npy1 mutants. Such analysis would be crucial to demonstrate that NPY1 is required for the observed phosphorylation events.

This is an excellent point and we agree with the reviewer that analyzing loss-of-function *npy* mutants is important. The challenge is that we need to knockout *NPY1*, NPY3, and NPY5 to phenocopy *pid*. We will also need to find a way to suppress the *npy* triple mutants, which are sterile, so that we can have meaningful comparisons.

(2) The functional consequences of the newly identified phosphorylation sites in PINs remain speculative. Site-directed mutagenesis (phospho-defective and phospho-mimetic) would help clarify their physiological roles.

We agree with the reviewer on this point as well. However, this is not trivial, as we have uncovered so many phosphorylation sites.

(3) The kinase responsible for NPY1-mediated phosphorylation remains unidentified. Since NPY1 is a non-kinase protein, a model involving recruitment of partner kinases (e.g., PIN-phosphorylating kinases other than PID) should be considered or discussed.

we will add a sentence to mention D6PK and other kinases in the Discussion in the revised version. We are hoping that the kinases will come out of future forward genetic screens.

**Reviewer #3 (Public review):**

Summary:

This manuscript from Mudgett et al. explores the relative roles of PID and NPY1 in auxin-dependent floral initiation in Arabidopsis. Micro vectorial auxin flows directed by PIN1 are essential to flower initiation, and loss of PIN1 or two of its regulators, PID and NPY1 (in a yucca-deficient background) phenocopies the pinformed phenotype. This group has previously shown that PID-PIN1 interactions and function are dosage-dependent. The authors pick up this thread by demonstrating that a heterozygote containing a CRISPR deletion of one copy of PIN1 can restore quasi-wild type floral initiation to pid.

The authors then show that overexpression of NPY1 is sufficient to more or less restore wild-type floral initiation to the pid mutant. The authors claim that this result demonstrates that NPY1 functions downstream of PID, as this ectopic abundance of NPY1 resulted in phosphorylation of PIN1 at sites that differ from sites of action of PID. The authors pursue evidence that PID action via NPY1 is analogous to the mode of action by which phot1/2 act on NPH3 in seedling phototropism. Such a model is supported by the evidence presented herein that the C terminus of NPY1, which has abundant Ser/Thr content, is phosphorylated, and that the deletion of this domain prevents overexpression compensation of the pinformed phenotype.

While the results presented support evidence in the literature that PID acts on NPY1 to regulate PIN1 function, it is also possible that NPY1 overexpression results in limited expansion of phosphorylation targets observed with other AGC kinases. And if the phot model is any indication, there may be other PID targets that modulate PIN1-dependent floral initiation.

However, overexpression of the NPY1 C-terminal deletion construct resulted in phosphorylation of both PIN1 and PIN2 and agravitropic root growth similar to what is observed in pin2 mutants. This suggests that direct PID phosphorylation of PINs and action via NPY1 can be distinguished by phosphorylation sites and by growth phenotypes.

Strengths:

A very important effort that places NPY1 downstream of PID in floral initiation.

We thank the reviewer for the comments.

Weaknesses:

As PID has been shown to act on sites that regulate PIN protein polarity as well as PIN protein function, it would be useful if the authors consider how their results would fit/not fit with a model where combinatorial function of NPY1 and PID regulate PIN1 in a manner similar to the way that PID appears to function combinatorially with D6PK on PIN3

We agree with the reviewer that we do not have a complete picture of how NPY, PID, PIN work together to control flower initiation. Some aspects of our results are difficult to reconcile with the model of PIN1 and PID acting in tandem, i.e., by PID directly phosphorylating and activating PIN1. Indeed, our results suggest that PIN1 and PID have opposite effects on organogenesis. For example, heterozygous pin1 (or PIN1-GFP_HDR,_ which is presumably less active than wild type PIN1) suppresses the pid phenotype. Moreover, pid and pin1 have opposite effects on cotyledon number and true leaf number. Mutations in PID lead to more cotyledons and more true leaves than WT whereas pin1 mutants make fewer cotyledons and fewer true leaves than WT (Bennett SRM, Alvarez J, Bossinger G, Smyth DR (1995) Morphogenesis in pinoid mutants of *Arabidopsis thaliana*. The Plant Journal 8: 505-520). We have elaborated on this point in the last paragraph of the Discussion.

The genetic materials we have generated may allow us to uncover additional components in the pathway from forward genetic screens, which may eventually lead to a clear picture.
